# Short-term dietary methionine supplementation affects one-carbon metabolism and DNA methylation in the mouse gut and leads to altered microbiome profiles, barrier function, gene expression and histomorphology

**DOI:** 10.1186/s12263-017-0576-0

**Published:** 2017-09-06

**Authors:** Isabelle R. Miousse, Rupak Pathak, Sarita Garg, Charles M. Skinner, Stepan Melnyk, Oleksandra Pavliv, Howard Hendrickson, Reid D. Landes, Annie Lumen, Alan J. Tackett, Nicolaas E.P. Deutz, Martin Hauer-Jensen, Igor Koturbash

**Affiliations:** 1 Department of Environmental and Occupational Health, University of Arkansas for Medical Sciences, 4301 W. Markham Str., Slot 820-11, Little Rock, AR 72205-7199 USA; 2 Department of Pharmaceutical Sciences, University of Arkansas for Medical Sciences, Little Rock, AR 72205 USA; 3 Department of Pediatrics, University of Arkansas for Medical Sciences, Little Rock, AR 72205 USA; 4 Department of Biostatistics, University of Arkansas for Medical Sciences, Little Rock, AR 72205 USA; 5 Division of Biochemical Toxicology, National Center for Toxicological Research, U.S. Food and Drug Administration, Jefferson, AR USA; 6 Department of Biochemistry, University of Arkansas for Medical Sciences, Little Rock, AR USA; 7 Department of Health and Kinesiology, Center for Translational Research on Aging and Longevity, Texas A&M University, College Station, TX USA

**Keywords:** *Burkholderiales*, Essential amino acid, Gut microbiome, LINE-1, Methionine toxicity, Tight junction-related proteins

## Abstract

**Background:**

Methionine, a central molecule in one-carbon metabolism, is an essential amino acid required for normal growth and development. Despite its importance to biological systems, methionine is toxic when administered at supra-physiological levels. The aim of this study was to investigate the effects of short-term methionine dietary modulation on the proximal jejunum, the section of the gut specifically responsible for amino acid absorption, in a mouse model. Eight-week-old CBA/J male mice were fed methionine-adequate (MAD; 6.5 g/kg) or methionine-supplemented (MSD; 19.5 g/kg) diets for 3.5 or 6 days (average food intake 100 g/kg body weight). The study design was developed in order to address the short-term effects of the methionine supplementation that corresponds to methionine dietary intake in Western populations. Biochemical indices in the blood as well as metabolic, epigenetic, transcriptomic, metagenomic, and histomorphological parameters in the gut were evaluated.

**Results:**

By day 6, feeding mice with MSD (protein intake <10% different from MAD) resulted in increased plasma (2.3-fold; *p* < 0.054), but decreased proximal jejunum methionine concentrations (2.2-fold; *p* < 0.05) independently of the expression of neutral amino acid transporters. MSD has also caused small bowel bacteria colonization, increased the abundance of pathogenic bacterial species *Burkholderiales* and decreased the gene expression of the intestinal transmembrane proteins—*Cldn8* (0.18-fold, *p* < 0.05), *Cldn9* (0.24-fold, *p* < 0.01) and *Cldn10* (0.05-fold, *p* < 0.05). Feeding MSD led to substantial histomorphological alterations in the proximal jejunum exhibited as a trend towards decreased plasma citrulline concentrations (1.8-fold, *p* < 0.07), as well as loss of crypt depth (by 28%, *p* < 0.05) and mucosal surface (by 20%, *p* < 0.001).

**Conclusions:**

Together, these changes indicate that short-term feeding of MSD substantially alters the normal gut physiology. These effects may contribute to the pathogenesis of intestinal inflammatory diseases and/or sensitize the gut to exposure to other stressors.

**Electronic supplementary material:**

The online version of this article (10.1186/s12263-017-0576-0) contains supplementary material, which is available to authorized users.

## Background

Methionine, a central molecule in one-carbon metabolism, is an essential amino acid required for normal growth and development [30, 31]. It is indispensable for protein synthesis and the production of polyamines [15]. Furthermore, methionine is a key regulator of stress resistance and is a precursor for S-adenosylmethionine (SAM), the principal donor of methyl groups, as well as cysteine and glutathione [16, 19, 46]. Studies using a neonatal piglet model have demonstrated the high importance of dietary methionine especially during early stages of life [8, 48, 63]. A number of studies report beneficial effects of methionine dietary supplementation on gut function, including improvement of the mucosal villus architecture, as well as methionine intake-associated decreased risk of colon cancer [7, 8, 49, 76]. Alterations to one-carbon metabolism and the methionine cycle are linked to a number of diseases, including cardiovascular disease and cancer [16, 22, 68].

Despite the importance of methionine to biological systems, it is by far the most toxic amino acid [10, 37]. The formation of methanethiol-cysteine disulfides is thought to cause methionine toxicity [10]; however, this alone cannot explain the wide range of health outcomes associated with an increased consumption of methionine, including growth retardation, infertility, accumulation of hemosiderin, inflammatory responses, liver damage, and cardiovascular disease (reviewed in [34]). Some recent studies indicate that methionine supplementation accelerates oxidative stress and activates *NFkB* in the mouse liver [59]. Aissa and colleagues have reported that, in the mouse model, methionine dietary supplementation increased hepatic levels of S-adenosyl-L-homocysteine and homocysteine, altered expression of one-carbon and lipid metabolism genes, and caused lipid accumulation in the liver [1].

Although liver is considered a major organ for methionine metabolism, it becomes increasingly recognized that the intestine also serves as a significant site of dietary methionine metabolism [7, 17, 63, 66]. However, the exact fate of dietary methionine in the proximal intestine, the section of the gut specifically responsible for amino acid absorption, remains to be investigated. Furthermore, the host-intestinal microbiome axis adds an additional layer of complexity, given the tight relationship that exists between the host’s and microbiome’s amino acid metabolism [2, 52, 57]. Moreover, it has been demonstrated that the production of xenometabolites is under the influence of the host’s diet [42, 43]. Therefore, the aim of this study was to investigate the effects of the short-term methionine dietary modulation on the proximal jejunum in a mouse model.

## Methods

### Animals and diets

Eight-week-old CBA/J male mice were purchased from Jackson Laboratory (Bar Harbor, ME, USA). The animals were housed at the University of Arkansas for Medical Sciences (UAMS) animal facility with a 12 h:12 h dark/light cycle. The experimental protocols were reviewed and approved by the Institutional Animal Care and Use Committee (IACUC) at UAMS. Animals were given a 1-week acclimation period before the experiment commenced receiving methionine-adequate diet (MAD). After that, animals were randomly divided into two groups where half of the animals continued receiving MAD (*n* = 10), while the second half of the animals was fed methionine-supplemented diet (MSD) (*n* = 10) for 3.5 or 6 consecutive days. The study design was developed in order to address the short-term effects of the methionine supplementation that correspond to methionine dietary intake in Western populations. Water and food were provided ad libitum. All diets were purchased from Envigo (Madison, WI, USA). The detailed composition and nutrient information for each diet are provided in Tables 1 and 2. Animals were monitored on a daily basis; food and water consumption and body weights were recorded daily.

**Table 1:** Nutritional characteristics of the methionine-adequate (MAD, TD.140520) and methionine-supplemented (MSD, TD.160241) diets used in the study

Formula	TD.140520 g/kg	TD. 160,241 g/kg
Sucrose	445.297	432.197
Corn starch	198.783	198.783
Corn oil	100	100
Cellulose	30	30
Mineral mix, AIN-76 (170915)	35	35
Calcium phosphate, dibasic	3	3
L-alanine	3.5	3.5
L-arginine HCl	12.1	12.1
L-asparagine	6.0	6.0
L-aspartic acid	3.5	3.5
L-cystine	3.5	3.5
L-glutamic acid	40.0	40.0
Glycine	23.3	23.3
L-histidine HCl, monohydrate	4.5	4.5
L-isoleucine	8.2	8.2
L-leucine	11.1	11.1
L-lysine HCl	18.0	18.0
L-methionine	6.5	19.5
L-phenylalanine	7.5	7.5
L-proline	3.5	3.5
L-serine	3.5	3.5
L-threonine	8.2	8.2
L-tryptophan	1.8	1.8
L-tyrosine	5.0	5.0
L-valine	8.2	8.2
Total amino acids	177.9	190.9
Vitamin mix, Teklad (40060)	10.0	10.0
Ethoxyquin, antioxidant	0.02	0.02

**Table 2:** Methionine-adequate (MAD; TD.140520) and methionine-supplemented (MSD; TD.160241) diets nutrition information (calculated values)

	TD.140520		TD.160241	
	% by weight	% kcal from	% by weight	% kcal from
Protein	15.3	15.1	18.4	17.9
CHO	63.3	62.6	61.9	60.2
Fat	10.0	22.3	10.0	21.9

### Tissue harvest

On days 3.5 and 6, animals were anesthetized with isoflurane (3% in oxygen) and retroorbital bleeding in EDTA-coated tubes was performed in order to obtain blood samples. Blood was centrifuged at 10,000×*g* for 2 min at room temperature. Plasma was collected, flash-frozen in liquid nitrogen, and stored at −80 °C for subsequent analyses. Anesthetized mice were euthanized by cervical dislocation and intestines were collected immediately for the metabolic, molecular, and immunohistochemical analyses.

### Analysis of methionine plasma concentrations

Blood was centrifuged immediately after animal bleeding, and serum was stored at −80 °C conditions. Plasma methionine concentrations were determined using the commercially available EZ:fast amino acid kit for physiological amino acids (Phenomenex; Torrance, CA, USA). Samples (50 μl) were first prepared for derivatization using a solid phase extraction step followed by a derivatization and liquid/liquid extraction. Derivatized amino acids were extracted into a mixture of chloroform:iso-octane (1:2). The top organic layer was removed and evaporated to dryness under a gentle stream of nitrogen at room temperature. The residue was reconstituted in 100 μl of mobile phase and injected (1 μl) onto the LC-MS/MS system. Analyte separation was achieved using a gradient elution profile provided with the EZ:fast kit on a 250 × 2.0 mm EZ:fast analytical column. The flow rate was 0.25 ml/min. The total run time was 17 min.

### Tissue determination of analytical components of methionine metabolism

Proximal jejunum samples were flushed with 1X PBS and flash-frozen to further determine levels of methionine, S-adenosylmethionine (SAM), S-adenosylhomocysteine (SAH), total and free homocysteine and homocystine, cysteine, cystine, as well as reduced (GSH) and oxidized (GSSG) glutathione using an HPLC-EC method, as previously described [50, 51].

### Nucleic acids extraction

RNA and DNA were extracted simultaneously from flash-frozen tissue using the AllPrep DNA/RNA extraction kit (QIAGEN, Valencia, CA, USA) according to the manufacturer’s protocol (including RNase and DNase digestion for DNA and RNA, respectively). DNA and RNA concentrations and integrity were analyzed by the Nanodrop 2000 (Thermo Scientific, Waltham, MA, USA). For DNA, only samples with the 260/280 ratios between 1.8 and 1.9 and the 260/230 ratios above 1.5 were considered for further molecular analyses. For RNA, only samples with the 260/280 ratios between 1.95 and 2.05 and the 260/230 ratios above 1.5 were considered for further molecular analyses.

### Analysis of intra-intestinal mRNA levels of neutral amino acid transporters

RNA was extracted as described above. cDNA was synthesized using the SuperScript reverse transcription kit (Life Technologies, Carlsbad, CA, USA) according to the manufacturer’s protocol. Quantitative real-time PCR (qRT-PCR) was performed with Taqman Universal Master Mix (Life Technologies) according to the manufacturer’s protocol. Primers were added at a final concentration of 5 μM (Additional file 1). Expression of mRNA targets was normalized to the internal control genes *Hprt* and *Gapdh* and expressed as fold change according to the ΔΔCt method.

### Analysis of LINE-1 DNA methylation

Recent advances in computational biology have led to classification of LINE-1 elements based on their evolutionary age and respective 5′-UTR sequences [67]. In this study, we assessed the DNA methylation status of seven LINE-1 elements that belong to evolutionary the youngest A-type promoter. LINE-1 families’ consensus sequences were obtained from the Genetic Information Research Institute (GIRI) Database: http://www.girinst.org/ [4]. Then, the 5′-UTRs of seven LINE-1 elements were analyzed using NEBcutter® (http://nc2.neb.com/NEBcutter2/). The five most frequent CpG sites that can be cleaved by the methylation-sensitive restriction enzymes (AciI, BstUI, HhaI, HpaII, and SmaI) were identified and individual RT-PCR assays for each LINE-1 element were developed and validated. Analysis of the LINE-1 DNA methylation was performed as previously described [62]. Briefly, 1 μg of genomic DNA was digested with 1 U of SmaI in 1X CutSmart buffer at 25 °C for 2 h. This was followed by a 16 h digestion at 37 °C in the presence of 1 U of the HpaII, HhaI, and AciI in 1X CutSmart buffer. The digestion was finalized by adding 0.5 U of BstUI in 1X CutSmart buffer for 4 h at 60 °C (New England Biolabs, Ipswich, MA, USA). Digested DNA was then analyzed by qRT-PCR on a ViiA 7 real-time PCR system (Applied Biosystems, Forrest City, CA, USA). DNA samples not digested with the restriction enzyme mix served as a positive control, while samples lacking the specific primers for DNA amplification and/or DNA template served as negative controls. The Ct was defined as the fractional cycle number that passes the fixed threshold. The Ct values were converted into the absolute amount of input DNA using the absolute standard curve method and further normalized towards readings from the respective to each LINE-1 element ORF1 region that lacks CpG sites. Assays for determination of 5′-UTR LINE-1 DNA methylation are provided in Additional file 1.

### Gene expression analysis of mRNA levels of tight junction-related proteins

RNA was extracted using the QIAGEN DNA/RNA extraction kit (QIAGEN) according to the manufacturer’s instructions. Mouse tight junctions PCR array (SA Biosciences, array #PAMM143Z) was used to analyze the expression of genes involved in the regulation of tight junction-related proteins according to the manufacturer’s protocol.

### Gram staining of the intestinal microbiota

The proximal jejunum slides were deparafinized by placing them in a 60 °C oven for 15 min. The slides were then placed in xylene for 5 min twice, followed by 3 min in 100% ethanol twice. The slides were then immersed in 90% ethanol for 3 min, then in 80% ethanol for 3 min. The slides were rinsed under tap water for 30 s and placed in deionized water for 30 min. The slides were blotted and then stained for 30 s with Gram stain (Gram stain for tissue kit, Sigma-Aldrich, St. Louis, MO, USA). The Gram stain was drained off and the slides rinsed by immersion in deionized water. Gram’s iodine was added to the slides for 5 min, then drained and rinsed by immersing in deionized water. The slides were thoroughly differentiated in absolute alcohol then rinsed again in deionized water before adding saffranin for 30 s. Slides were drained and rinsed in deionized water and then blotted. Finally, tartrazine solution was added for 10 s then blotted. The slides were rinsed twice in 100% ethanol for 2 min, then in xylene for 2 min. The cover slip was mounted with permount (Fisher Scientific, Pittsburgh, PA, USA), and the slides were allowed to dry before microscopy.

### Analysis of the 16S rRNA in the proximal jejunum

Section of the proximal jejunum was cut with the sterile pair of scissors and flash-frozen in liquid nitrogen. DNA was extracted under sterile conditions as described above. Amplification of the bacterial 16S DNA gene was performed from 5 ng of the proximal jejunum gDNA using the following set of primers Fw: ACTCCTACGGGAGGCAGCAGT and R: TATTACCGCGGCTGCTGGC [20].

### Next generation sequencing of gut microbiota

Total intestinal DNA was extracted using the DNeasy Blood and Tissue Kit (QIAGEN). The extracted DNA analyzed by NanoDropTM 2000 (ThermoScientific) and 1% agarose gel electrophoresis (in TBE 0.5 X) and sent to Research and Testing Laboratories for 16S ribosomal RNA gene sequencing using the Illumina MiSeq System (Research and Testing Laboratories, Lubbock, TX, USA) as described before [41, 64].

16S rRNA genes were amplified by universal primers 357wF: CCTACGGGNGGCWGCAG and 785R: GACTACHVGGGTATCTAATCC using the Qiagen HotStarTaq Master Mix (Qiagen). Next generation sequencing was performed on the MiSeq platform (Illumina). Samples were amplified for sequencing in a two-step process. Primers for the first step were constructed using 357F-785R with the Illumina i5 and i7 sequencing primers added to the 5′-end of each, respectively. Products from the first amplification were added to a second Polymerase Chain Reaction (PCR) step based on qualitatively determined concentrations (amplicons were run on 2% ethidium bromide gel, gel bands were scored, and a volume of products was added to the second PCR based on the scores). Primers for the second PCR step were designed using Illumina Nextera PCR primers with 8 bp dual indices. Amplification products were then visualized with eGels (Life Technologies). After that, the products were pooled equimolar and each pool was then size-selected in two rounds using Agencourt AMPure XP (BeckmanCoulter) in a 0.7 ratio for both rounds. Size-selected pools were then quantified using the Qubit 2.0 florometer (Life Technologies) and loaded on an Illumina MiSeq 2 × 300 flow cell at 10 pM.

After sequencing, all failed sequence reads, low-quality sequence ends, tags and primers as well as any non-bacterial ribosome sequences and chimeras were removed using the UCHIME chimera detection software in de novo mode [27]. To curate the short (b150 bp) reads, sequences with ambiguous base calls, and sequences with relatively long homopolymers (N6 bp) were also removed. To determine the identity of bacteria in the remaining sequences, sequences were denoised, assembled into OTU clusters (97% identity) using the UPARSE algorithm [26], and then globally aligned using the USEARCH global algorithm [25] against a database of high-quality 16S rRNA bacterial gene sequences compiled by RTL Genomics (Lubbock, TX) to determine taxonomic classifications. After OTU selection was performed, a phylogenetic tree was constructed in Newick format from a multiple sequence alignment of the OTUs done in MUSCLE [23, 24] and generated in FastTree [60, 61]. Based upon the generated OTU table and taxonomy file, the bacteria were classified at the appropriate taxonomic levels. The percentages of sequences assigned to each bacterial phylogenetic level were individually analyzed for each pooled sample providing relative abundance information within and among the individual samples. All data have been uploaded to the publically available database https://www.ncbi.nlm.nih.gov/bioproject/PRJNA397387.

### Analysis of citrulline plasma concentrations

Whole blood was collected from the retroorbital sinus into EDTA-coated tubes (Fisher Scientific). Plasma was obtained by centrifugation at 12,000 RPM for 5 min at 4 °C and stored at −80 °C for further analysis. Citrulline plasma levels were determined using the high throughput liquid chromatography-tandem mass spectrometry (LC-MS/MS) methodology, as previously described [32, 36]. Briefly, plasma samples (10 μl) were treated with 490 μl acetonitrile:water:formic acid (85:14.8:0.2 *v*/*v*) containing internal standard (2 μM). After mixing gently, the samples were covered, allowed to stand for 5 min, and the filtrate was collected under vacuum.

### Intestinal crypt colony assay

Microcolony crypt cell survival was performed as previously described [11, 75]. Briefly, groups of mice fed either MAD or MSD diets were humanly euthanized on days 3.5 and 6, segments of proximal jejunum were obtained, fixed, and H&E stained. Surviving crypts, defined as crypts containing 10 or more adjacent chromophilic non-Paneth cells, were counted in transverse cross-sections. Four circumferences were scored per mouse and microcolony survival was expressed as the average number of crypts per circumference, with the average from each mouse considered as a single value for statistical purposes.

### Mucosal surface area analysis

Previously, our laboratory measured mucosal surface area in vertical sections using a stereologic projection/cycloid method as described by Baddeley et al. [3] and was adapted by us to our model system [47]. The method does not require assumptions about the shape or orientation of the specimens and thus circumvents problems associated with most other procedures for surface area measurement. Using the same principle, we developed an automated software to measure intestinal mucosal surface area in vertical H&E–stained sections of the jejunum by using a computer-assisted image analysis program (Image-Pro Premier, Meyer Instruments Inc., Houston, TX, USA). All measurements were done with a 10× objective lens and a total of three to five areas were measured from each intestinal segment.

### Statistical analysis

All data are presented as mean ± standard error of mean(s). Statistically significant differences for each treatment compared to the control (at *α* = 95%) were assessed using Student’s *t* test. Statistical analyses were performed using GraphPad Prism 6 (GraphPad Software Inc. LaJolla, CA, USA).

## Results

### Effects of MSD on animal body weight and food and water consumption

Feeding mice MSD initially led to small losses in the body weights; however, at the end of the experiment (day 6), the difference between the mice fed MSD and MAD was not detectable (Additional file 2). Feeding MSD did not affect significantly food or water consumption throughout the experiment (data not shown).

### MSD causes imbalance between the methionine tissue and plasma concentrations

**Fig. 1:**
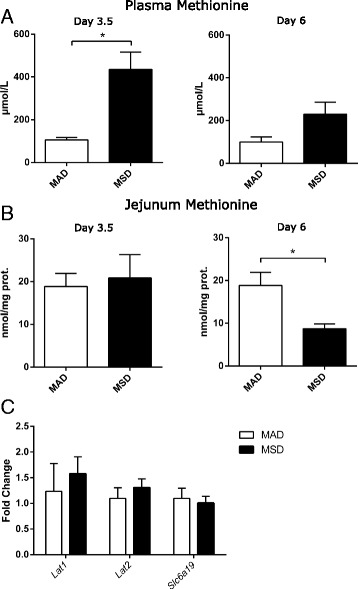
Methionine (**a**) plasma and (**b**) proximal jejunum concentrations in the mice fed methionine-adequate and methionine-supplemented diets. (**c**) Analysis of mRNA levels of three major neutral amino acid transporters in the proximal jejunum [*n* = 5 mice/group, asterisk “*” denotes significant (*p* < 0.05) difference from control]

Mice fed MSD were characterized by significantly elevated plasma methionine concentrations. Specifically, at day 3.5, methionine plasma concentrations were 4.2-fold (*p* < 0.05) higher in the mice fed MSD compared to the mice fed MAD. Methionine plasma concentrations remained elevated at day 6 although to a lower extent (2.3-fold, *p* < 0.054) (Fig. 1a). At the same time, the proximal jejunum methionine tissue concentrations did not increase at day 3.5 and were substantially decreased (0.45-fold, *p* < 0.05) at day 6 (Fig. 1b).

### MSD does not affect the expression of neutral amino acid transporters

In order to investigate the mechanisms of the imbalance between the plasma and intestinal methionine concentrations, first we analyzed the expression of a number of genes, implicated in the transport of neutral amino acids, and methionine in particular—*Lat1*, *Lat2*, and *Slc6a19* [14]. We did not identify any significant differences in the expression of those genes between the mice fed MAD and MSD (Fig. 1c).

### MSD-induced changes in the intestinal microbiome

A number of recent studies demonstrated that shifts in the gut microbiome may affect the levels of circulating blood metabolites [73]. In addition, methionine has been generally recognized as a key molecule in the metabolism of the intestinal flora [40]. Therefore, we then addressed the effects of MSD on the mouse intestinal microbiota.

**Fig. 2:**
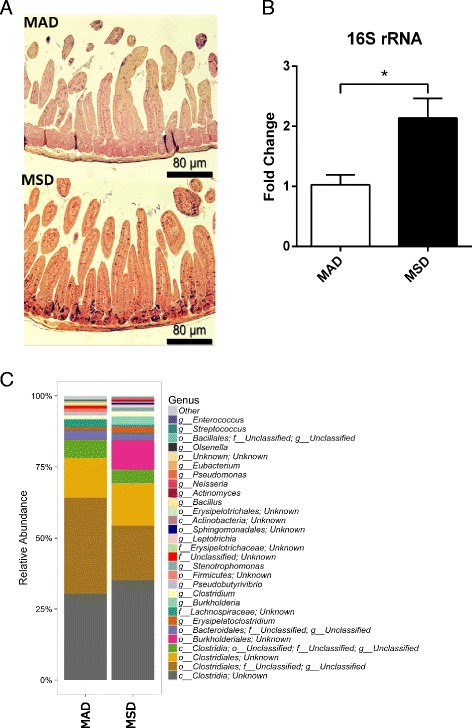
Methionine dietary supplementation leads to (**a**) bacterial proliferation in the mouse proximal intestine: MAD - Gram stain of the proximal jejunum of a mouse fed methionine-adequate diet, MSD – Gram stain of the proximal jejunum of a mouse fed methionine-supplemented diet; (**b**) increase in bacterial DNA in the proximal jejunum, and (**c**) shifts in the intestinal microbiome as analyzed by the 16S rRNA gene sequencing

Feeding mice a diet with the excess methionine for 6 days stimulated bacterial proliferation in the murine proximal jejunum (Fig. 2a). We have confirmed these findings with the 16S rRNA-based qPCR, where the significant (2.1-fold, *p* < 0.05) increase in the proximal jejunum of mice fed MSD was observed (Fig. 2b).

The next generation sequencing of the gut microbiome was done with the sample size of 3/group and returned an average of 20,562 classified reads per sample. There was an average of 127 observed OTU per sample. The PCA analysis showed a complete separation between the MAD and MSD groups. Axis 1 accounts for half of the variation, and axis 2 accounts for 27% (Additional file 3). The rarefaction plot of species richness, Chao1 richness, and Shannon diversity all pointed to a decrease in the number of species detected in MSD compared to MAD (Additional file 4). Furthermore, MSD caused substantial shifts in the intestinal microbiome at both the phylum and lower taxonomic levels. Specifically, MSD significantly decreased the proportion of firmicutes in comparison to MAD. At the lower taxonomic levels, MSD induced a significant decrease in *Clostridiales*, which are heavily involved in intestinal metabolism, and concomitantly increased the abundance of *Burkholderiales.* (Fig. 2c).

### MSD causes substantial changes in the expression of markers of intestinal inflammation and tight junctions-related proteins

Shifts in intestinal microbiota profiles may cause intestinal inflammation and affect the expression of tight junction-related proteins leading to increased intestinal permeability [18, 33, 69]. Furthermore, modulation of the methionine dietary intake has recently been reported to affect the expression of the intestinal tight junction-related proteins [56]. Gene expression analysis revealed a non-significant trend towards increase (2.6-fold, *p* < 0.2) in the expression of *Tnfα* in the proximal jejunum of mice fed MSD, compared to MAD-fed mice on day 6. Furthermore, a number of tight junction-related proteins were found to be differentially regulated as a result of MSD. Specifically, feeding mice MSD decreased the expression of the transmembrane proteins—claudins *Cldn8* (0.18-fold, *p* < 0.05), *Cldn9* (0.24-fold, *p* < 0.01), and *Cldn10* (0.05-fold, *p* < 0.05) (Table 3). Additionally, MSD caused decreases in the expression of G-protein signaling gene *Gnai1* (0.36-fold, *p* < 0.05) and protein kinase *Mpp5* (0.51-fold, *p* < 0.05).

**Table 3:** Altered expression of tight junction-related genes in the mouse proximal jejunum after feeding methionine-supplemented diet (fold-change relative to MAD)

Gene	Fold change relative to MAD	95% CI
*Cldn8*	0.18*	(0.03, 0.33)
*Cldn9*	0.24**	(0.12, 0.36)
*Cldn10*	0.05*	(0.001, 0.11)
*Gnail1*	0.36*	(0.13, 0.59)
*Mpp5*	0.51*	(0.23, 0.79)

*MAD* methionine-adequate diet. *p < 0.05; **p < 0.01

### MSD causes alterations in the methionine cycle and DNA methylation

Because methionine is a key molecule in the one-carbon metabolism and methionine cycle particularly, alterations in tissue methionine concentrations may subsequently affect all downstream metabolites. Indeed, MSD significantly affected not only the intestinal tissue methionine concentrations, but the entire methionine cycle. While at day 3.5, only small changes were observed and limited primarily to increased levels of SAH and cysteine and subsequently SAM/SAH and cysteine/cystine ratios, more pronounced changes were observed at day 6 (Table 4). At this time-point, mice fed MSD were characterized by substantially (over twofold) decreased levels of intra-intestinal methionine, followed by decreased concentrations of SAM, cysteine, and GSH, leading to skewed SAM/SAH, cysteine/cystine and GSH/GSSG ratios.

**Table 4:** Alterations in the proximal jejunum methionine cycle as a consequence of administration of the methionine-supplemented diet (MSD)

	Day 3.5		Day 6	
	MAD	MSD	MAD	MSD
Methionine, nmol/mg prot	18.86 ± 3.02	20.83 ± 5.47	18.86 ± 3.02	8.74 ± 1.09*
Total homocysteine, nmol/mg prot	0.75 ± 0.05	0.76 ± 0.05	0.75 ± 0.05	0.65 ± 0.05
Free homocysteine, nmol/mg prot	0.18 ± 0.01	0.18 ± 0.02	0.18 ± 0.01	0.20 ± 0.01
Homocystine, nmol/mg prot	0.23 ± 0.02	0.21 ± 0.02	0.23 ± 0.02	0.18 ± 0.01*
Homocystine/Free homocysteine, ratio	1.27 ± 0.09	1.19 ± 0.03	1.27 ± 0.09	0.86 ± 0.03*
SAM, nmol/mg prot	2.13 ± 0.36	1.94 ± 0.31	2.13 ± 0.36	1.63 ± 0.08*
SAH, nmol/mg prot	0.36 ± 0.02	0.51 ± 0.10*	0.36 ± 0.02	0.41 ± 0.02
SAM/SAH, ratio	5.82 ± 0.71	3.97 ± 0.43*	5.82 ± 0.71	4.01 ± 0.37*
Cysteine, nmol/mg prot	12.66 ± 2.14	8.85 ± 1.44	12.66 ± 2.14	6.42 ± 0.60*
Cystine, nmol/mg prot	1.06 ± 0.05	1.29 ± 0.06*	1.06 ± 0.05	1.49 ± 0.27*
Cysteine/Cystine, ratio	12.29 ± 2.65	6.89 ± 1.06*	12.29 ± 2.65	4.55 ± 0.39*
GSH, nmol/mg prot	31.00 ± 4.21	27.75 ± 3.41	31.00 ± 4.21	21.92 ± 1.38*
GSSG, nmol/mg prot	0.73 ± 0.07	0.76 ± 0.06	0.73 ± 0.07	0.87 ± 0.05
GSH/GSSG, ratio	42.91 ± 5.17	36.57 ± 3.50	42.91 ± 5.17	25.62 ± 2.58*

*MAD* methionine-adequate diet; *p < 0.05

Altered levels of SAM may further affect the process of DNA methylation, since SAM serves as a primary donor of methyl groups. By covering ~20% of mammalian genomes, LINE-1 elements are the most abundant transposable elements in the living organisms whose expression is under a tight control of DNA methylation [53]. Therefore, their methylation status has generally been recognized as a surrogate marker for global DNA methylation status [54].

**Fig. 3:**
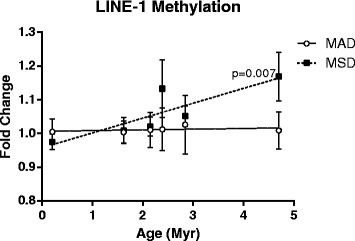
Methionine dietary supplementation affects DNA methylation of LINE-1 elements in the evolutionary age-dependent manner. Data are presented as mean ± SEM (*n* = 5) and show the linear regression between DNA methylation and evolutionary age of the element

Here, we identified that administration of MSD significantly shifted the patterns of DNA methylation of LINE-1 elements in the mouse proximal jejunum (Fig. 3). This effect was dependent on the evolutionary age of the elements, where the evolutionary younger (hence, more methylated [62]) LINE-1 elements were hypomethylated, while the evolutionary elder LINE-1 elements showed tendency towards DNA hypermethylation (*r* = 0.1483, *p* = 0.007).

### MSD affects normal intestinal histomorphology

Because of the substantial alterations to one-carbon metabolism and the methionine cycle, we hypothesized that these changes may result in impaired protein synthesis and thus result in decreased enterocyte renewal properties of the mouse gut.

**Fig. 4:**
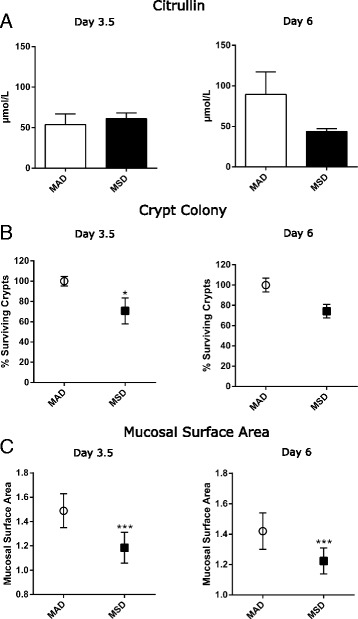
Methionine-supplemented diet affects histomorphology of the mouse proximal jejunum. (**a**) Analysis of the citrulline plasma concentrations (*n* = 5 mice/group). (**b**) Mucosal surface analysis. (**c**) Crypt colony assay (*n* = 10 mice/group). Asterisks “*” denotes significant (*p* < 0.05) and “***” (*p* < 0.001) difference from control

Citrulline is a non-coding amino acid and an end-product of proximal jejunum-associated enterocytes. Therefore, plasma concentrations of citrulline serve as a well-validated surrogate biomarker for the functional enterocyte mass [9, 21]. By day 6 of feeding mice MSD, the trend towards decreased plasma citrulline concentrations was identified (1.8-fold, *p* < 0.07) when compared to mice fed MAD (Fig. 4a). This finding may suggest a decrease in the proximal jejunum total enterocyte mass.

Indeed, decreased methionine tissue concentrations that lead to impaired protein synthesis have substantially affected the normal intestinal histomorphology. As evident from Fig. 4, a ~ 30% decrease in the functional crypts was observed in mice fed MSD by day 6. Similarly, MSD substantially decreased the mucosal surface area in the mouse proximal jejunum compared MAD-fed mice at both time-points (by 20%, *p* < 0.001) (Fig. 4c).

## Discussion

Accumulating evidence indicates that the essential amino acid methionine may exert a certain degree of toxicity when administered at supra-physiological levels [10, 37, 65]. At the same time, due to fortification of grains and high supplemental levels, consumption of the methyl donor pathway components, including methionine, substantially grew in recent decades [65]. Furthermore, given that the consumption of supra-physiological amounts of methionine is common in Western populations [35, 68] and the fact that methionine has been proposed as a prospective radiomitigator [5, 6], investigating the potential methionine-induced gastrointestinal toxicity is highly important.

In this study, we aimed to examine the effects of the short-term feeding of MSD on the mouse proximal jejunum. This section of the gut serves as a primary site of amino acid absorption and is characterized by the high rates of cellular proliferation and intense protein renewal [12]. The diet used in our study has been chosen because its methionine concentration (threefold over the methionine concentration in the MAD) is considerably translational to the 2–3.3-fold increased protein methionine intake in current Western populations [35, 68]. This diet has been utilized extensively in the past and is not known to cause significant toxicity in the murine models.

In our mouse model, MSD had a transitory effect on the body weight, leading to slightly decreased weights on days 2-4, without significant changes in the food or water intake between the MSD and MAD fed animals. This may be potentially explained by the physiological adaptation to the new diet.

Feeding mice MSD has led to an unexpected loss of the methionine intra-intestinal tissue concentrations, while the plasma methionine concentrations were elevated throughout the study. This paradoxical imbalance between the plasma and tissue concentrations was not due to the impaired function of the neutral amino acid transporters, as their mRNA abundance remained unchanged. Although we cannot exclude the post-transcriptional inhibition of the transporters, it is very unlikely that a nearly twofold decrease in the intra-intestinal methionine concentrations is caused by a substantially decreased transport, given a four-fold increase in the methionine plasma concentrations at the same time-points.

To further explore this phenomenon, we first sought to investigate the effect of MSD on the gut microbiome. Methionine is of particular importance for bacteria that are capable of accumulating methionine against a concentration gradient, use evolutionarily acquired diverse methionine biosynthesis pathways, and are significant contributors to their host’s methionine metabolism [28, 40]. An increase in the dietary methionine intake would subsequently result in an increased amount of methionine in the lumen and provide an excellent source of fuel for rapid bacterial proliferation that was confirmed in our study by the intra-intestinal Gram staining. Because the Gram staining is strongly biased towards Gram-positive bacteria, we have confirmed these findings by analysis of the 16S rRNA in the proximal jejunum of mice. Furthermore, MSD has led to substantial shifts in the intestinal microbiome.

Intestinal barrier function is critical for maintaining not only the gut health, but the normal function of the entire organism. Tight junction-related proteins play a central role in this barrier function controlling the intestinal permeability [18, 69]. Observed in our study, the MSD-induced dramatic loss in the expression of transmembrane proteins (claudins) suggests the disruption of the intestinal barrier that can lead to the paracellular transport of water, electrolytes, and amino acids, as well as bacterial translocation and development of chronic inflammation. Furthermore, it is becoming increasingly recognized that the increase in intestinal permeability, mediated by altered function of the tight junction-related proteins, plays a critical role in the pathogenesis of numerous diseases, including inflammatory bowel disease, irritable bowel syndrome, and celiac disease (reviewed in [18]).

Decreased tissue concentrations of methionine have inevitably led to alterations in one-carbon metabolism pathways. A number of down-stream metabolites in the methionine cycle were affected, including SAM, cysteine, and glutathione. Of particular interest are the decreased levels of SAM paralleled by the increased levels of SAH that have resulted in a skewed SAM/SAH ratio with the shift towards SAH. This probably led to SAM’s decreased ability to donate its methyl groups for DNA methylation, which is of critical importance in rapidly proliferating organs such as the proximal jejunum. We used the methylation status of the 5′-UTR of LINE-1 elements as a measurement of DNA methylation. Given the overall genomic abundance of LINE-1 elements that cover ~20% of the mammalian genomes, their methylation status is generally considered as a surrogate biomarker of DNA methylation [54]. Furthermore, various environmental stimuli may adversely affect the DNA methylation status of LINE-1 [53, 55]. Because substantial differences may be detected between the methylation status of different LINE-1 elements based on their evolutionary age [45, 62], we have addressed the DNA methylation in the 5′-UTRs of six LINE-1 families that substantially differ by their evolutionary age (from 0.21 MYR to 4.33 MYR). Interestingly, small losses in DNA methylation were observed in the evolutionary the youngest LINE-1 element (L1MdA_I) that was previously characterized as the most methylated among the LINE-1 elements [62]. At the same time, DNA hypermethylation was observed in the evolutionary older elements, reaching its maximum in the L1MdA_VI element. It has been shown that the loss of DNA methylation occurs primarily from the evolutionary younger elements, while DNA hypermethylation occur primarily at the older, more demethylated elements as a result of exposure to ionizing radiation [45, 55, 62]. While the loss of L1MdA_I DNA methylation may be explained by the decreased SAM availability to donate its methyl groups, the DNA hypermethylation phenomenon in the older elements would require further investigation.

Feeding mice MSD induced significant structural alterations in the intestinal wall which exhibited as decreased intestinal mucosal surface length, diminished crypt depth, and trends towards reduced plasma concentrations of the enterocyte metabolic end-product citrulline. The most plausible explanation to this seems to be a dramatic decrease in the intra-intestinal methionine tissue concentrations. Methionine is known to be the first amino acid from which the protein synthesis starts from [30] and the lack of “building blocks” would inevitably decrease the intestinal cell wall renewal potential.

It is crucial to identify the specific mechanisms of the paradoxical loss of the methionine tissue concentration. To our knowledge, no studies reported this effect before. For instance, Finkelstein and Martin reported that even a 10-fold increase in the methionine dietary intake did not affect the hepatic methionine concentrations during the 7-day course of the study [31]. It must be noted that although liver is primarily a mitotically dead organ in which regeneration occurs only in the case of substantial injury, the intestinal mucosa undergoes rapid regeneration with a total cell wall renewal within the ~3.5 day course. This predetermines very intense protein renewal. Previous studies demonstrated that chronic inflammatory diseases, infections, and nutritional status (including particular amino acids) may significantly modulate intestinal protein metabolism [12, 13, 71, 74]. The intestinal inflammation and bacterial proliferation with an abundance of pathogenic species observed in our study could indeed lead to increased protein turnover. Another possible explanation is the lack of methionine availability to enterocytes. The latter primarily utilizes the apical transport directly from the lumen [12]. In these regards, the amount of methionine available to enterocytes may be substantially compromised: it might be either rapidly captured by the boosting microbiome or swiftly transported from the lumen into the blood paracellulary. Future studies are clearly needed to confirm or rule out these hypotheses. Furthermore, studies utilizing other mouse strains will be needed to investigate the genotype-dependent nature of methionine metabolism.

The effects caused by methionine dietary supplementation may potentiate the effects of various exogenous and endogenous stimuli. For instance, it has recently been shown that excessive methionine dietary intake potentiates ethanol-induced oxidative stress and dyslipidemia in rats [44]. Another study demonstrated that even moderate increases in methionine dietary intake are atherogenic in susceptible mice [70]. Furthermore, to our knowledge, no studies have investigated the effects of methionine dietary intake in combination with gastrointestinal injury inducers. Therefore, the effects of ionizing radiation that causes substantial mucosal stem cell death, leading to enterocyte depletion are particularly interesting [38, 72]. Further, bacterial infection is one of the leading causes of death, as well as the major driver of the acute radiation-induced gastrointestinal toxicity as a result of accidental exposure or radiotherapy [29, 38, 39, 58]. In these regards, the compromised gut with already affected permeability, the abundance of pathogenic bacteria, and depleted enterocyte mass may be predisposed to more severe acute radiation toxicity. These studies are underway in our laboratories and will be reported elsewhere.

## Additional files

Additional file 1: Gene expression and LINE-1 DNA methylation assays used in the study. (DOCX 16 kb)

Additional file 2: Body weight dynamics of mice fed methionine-adequate (MAD) and methionine-supplemented (MSD) diets. (PDF 54 kb)

Additional file 3: Gut microbiome analysis in mice fed methionine-adequate and methionine-deficient diets. Principal Coordinates Analysis based on unweighted Unifrac distances. (EPS 345 kb)

Additional file 4: Gut microbiome analysis in mice fed methionine-adequate and methionine-deficient diets, colored by group. A. Rarefaction plot of species richness, subsampling from 500 to 20,000 reads in increments of 500 reads. B. Chao1 richness within the total microbiome data showing the mean value (and confidence interval) in each group. C. Shannon diversity within the total microbiome data showing the mean value (and confidence interval) in each group. (EPS 3776 kb)

## Abbreviations

LINE-1: Long interspersed nucleotide element 1
MAD: Methionine-adequate diet
MSD: Methionine-supplemented diet
MYR: Million years
NFkB: Nuclear factor kappa B
ORF: Open reading frame
OUT: Operational taxonomic unit
qRT-PCR: Quantitative real-time polymerase chain reaction
rRNA: Ribosomal RNA
SAH: S-adenosylhomocysteine
SAM: S-adenosylmethionine
UTR: Untranslated region
