# Supplementary material for: Short-term dietary methionine supplementation affects one-carbon metabolism and DNA methylation in the mouse gut and leads to altered microbiome profiles, barrier function, gene expression and histomorphology
Source: Genes Nutr. 2017 Sep 6;12:22. doi: 10.1186/s12263-017-0576-0 (PMC5588631; doi:10.1186/s12263-017-0576-0)
Supplement: Supplementary file 1 — Gene expression and LINE-1 DNA methylation assays used in the study. (DOCX 16 kb) [file 12263_2017_576_MOESM1_ESM.docx]

**Additional file 1.** Gene expression and LINE-1 DNA methylation assays used in the study.

Gene expression assays

| **Target** | **ID** | **Source** |
| --- | --- | --- |
| *Gapdh* | Mm99999915_g1 | Life Technologies |
| *Lat1* | Mm.PT.58.10563809 | Integrated DNA Technologies |
| *Lat2* | Mm.PT.58.13740413 | Integrated DNA Technologies |
| *Slc6a19* | Mm.PT.58.11098212 | Integrated DNA Technologies |
| *Tnf* | Mm.PT.56a.12575861 | Life Technologies |

LINE-1 5’-UTR DNA methylation assays

| **LINE-1 Family** | **Forward Primer** | **Reverse Primer** |
| --- | --- | --- |
| L1MdA_I | AGTGGATCACAGTGCCTGC | GGGTAGCCTGCTTCCCTATG |
| L1MdA_II | ACCTCTGGTGAGTGGAACAC | CAAGACTCTGCTGGCAAGGTA |
| L1MdA_III | AAGTCCCTTCCGCTCGACTC | GTACCGCAGTCTCAGGTTCC |
| L1MdA_V | GGCCCACTGCAGCACC | TTCCTTAATTAATGCAGTCTCAGGT |
| L1MdA_VI | AAGTCCCTTCCGGTCCACTC | GCTTCCCTAGTTAATGCAGTCTC |
| L1MdA_VII | CCTCTGGTGAGTGGAACACA | CTTAAGATCCCGTGGAGGGTC |
