# Supplementary figures and images for: Short-term dietary methionine supplementation affects one-carbon metabolism and DNA methylation in the mouse gut and leads to altered microbiome profiles, barrier function, gene expression and histomorphology
Source: Genes Nutr. 2017 Sep 6;12:22. doi: 10.1186/s12263-017-0576-0 (PMC5588631; doi:10.1186/s12263-017-0576-0)

## Body weight

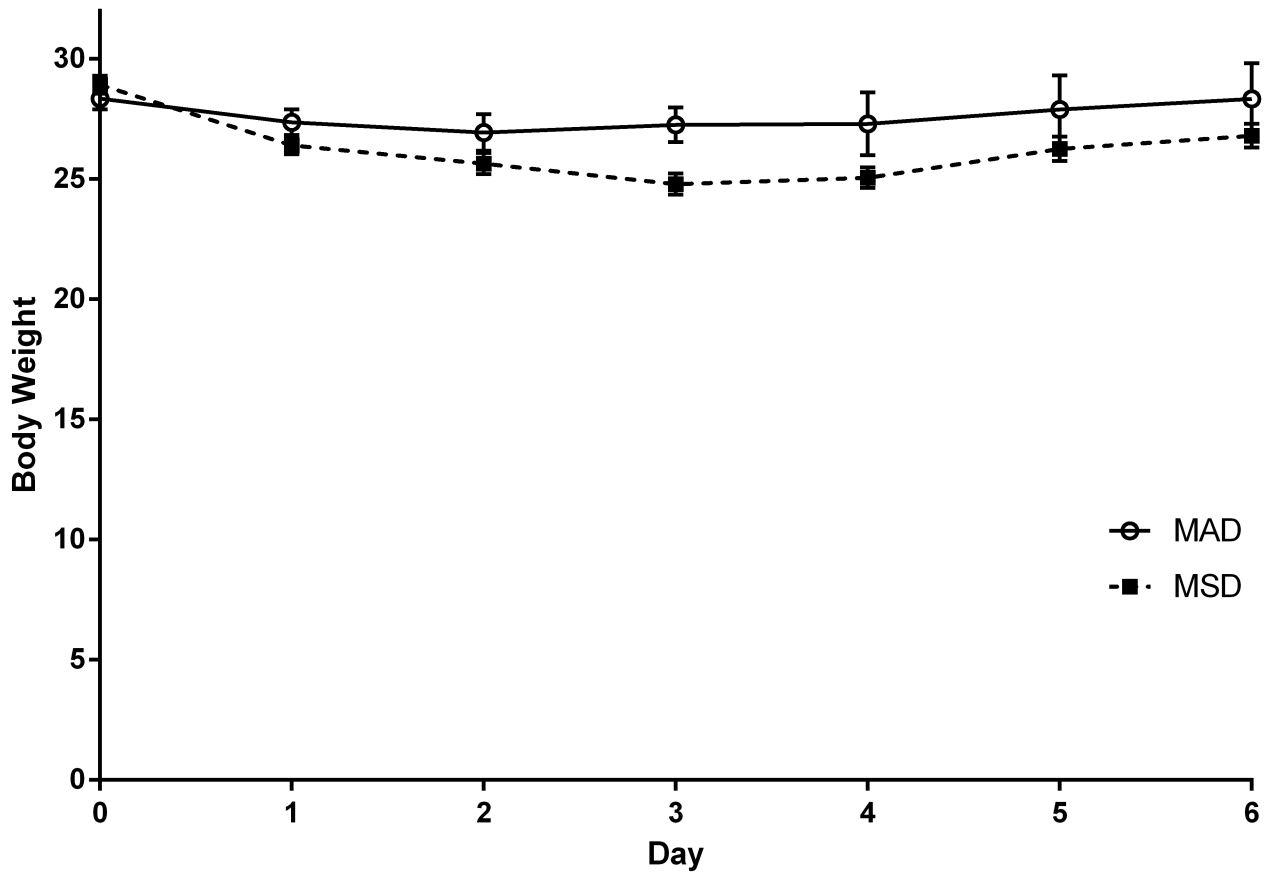

Supplement: Supplementary file 2 — Body weight dynamics of mice fed methionine-adequate (MAD) and methionine-supplemented (MSD) diets. (PDF 54 kb) [file 12263_2017_576_MOESM2_ESM.pdf]
